# The immunopathogenesis of cow’s milk protein allergy (CMPA)

**DOI:** 10.1186/1824-7288-38-35

**Published:** 2012-07-23

**Authors:** Vitaliti Giovanna, Cimino Carla, Coco Alfina, Praticò Andrea Domenico, Lionetti Elena

**Affiliations:** 1 Bronchopneumoallergology and Cystic Fibrosis O.U., Departement of Pediatrics, University of Catania, AOU Policlinico-OVE, Via Santa Sofia 78, 95123 Catania, Italy

## Abstract

The most frequent symptoms among the manifestations of cow milk protein allergy (CMPA) are gastrointestinal. CMPA pathogenesis involves immunological mechanisms with participation of immunocompetent cells and production of immunoglobulin E (IgE). Nevertheless, recent studies have been focused on the description of other forms of CMPA, not-mediated by IgE reactions, mostly involving the T lymphocite immune system. Thus, in this field it is important to note how different kind of cells are involved in the immunopathogenesis of CMPA, such as antigen-specific T cells, T regulatory cells, cytokines secreted by the different T lymphocite subsets, B lymphocytes, antingen-presenting cells, mast cells, that together orchestrate the complex mechanism leading to the phenotipic expression of CMPA.

The progress in the diagnosis of immunologic disorders allowed the recent literature to develop new models for immuno-mediate disorders, involving new cells (such as Treg cells) and thus allowing the acquisition of a new vision of the pathogenesis of atopic diseases.

The aim of this review is to describe the immunopathogenetic aspects of CMPA in view of these new discoveries in the immunologic field, considering the immunologic pathway at the basis of both IgE- and not-IgE mediated CMPA.

## Introduction

The main role of the human gastrointestinal tract is the reduction of ingested food in simple elements that can be absorbed and used for energy production and cell growth.

In order to prevent an indiscriminate immunization, secondary to the absorption of foreign antigens through the gastrointestinal barrier, the gut has developed non specific (non-immunological) mechanisms [1], such as the intestinal mucosal barrier, the intestinal motility, secretion of mucus, gastric acidity, enzymes, and specific (immunological) factors, such as the production of secretory IgA and antigen interaction with the Gut Associated Lymphoid Tissue (GALT) [2]. As a matter of fact, in normal individuals, antigen presenting cells, mostly dendritic cells sited in the GALT, play a main role in the development of a tolerogenic response. They process the food antigen and present it on a major histocompatibility complex (MHC) class II receptor to the T cells, resulting physiologically in a status of imunologic homeostasis known as “oral tolerance” by deletion or inhibition of antigen-specific T cells and production of regulatory T cells (Treg) that suppress inflammatory responses to antigens [3,4].

The pathogenic mechanism of the mucosal tolerance has not yet clarified. Recent studies have suggested that human enterocytes could play a key role capturing soluble antigens and selectively activating CD 8+ T cells with a suppressive function [5].

Another explanation could be referred to a temporary dysfunction of the protective mechanisms above described, with a loss of tolerance and sensitization to food antigens. As a matter of fact, it has been suggested that enterocytes regulate the speed and the kind of absorption of ingested antigens. On this regard it has been illustrated that mucus has a major role as a barrier to foreign antigens, and food proteins reaching the gut are partially digested by proteases and by gastric acidity so that reduced gastric acidity in infants and intake of proton pump inhibitors may play a role in the pathogenesis of food allergy [6,7].

Approximately 50% of the protein absorption takes place in the duodenum, even though the whole small bowel is involved. These protein antigens may cross the epithelial barrier by transcytosis through enterocytes or by uptake via the Microfold cells (M-cells) [8,9]. Food proteins may diffuse paracellularly through the epithelial layer. In this case, the enhanced permeability is probably the result of the action of several proinflammatory cytokines [10]. In these cases, food proteins reach the MALT in large quantities, most often leading to IgG induction and immune complexes. So, the food-adverse reactions may be immunologic, but not IgE-mediated [11].

There are literature studies evidencing that in genetically predisposed individuals sensitization of naive T cells will lead to a TH2 type response with secretion of cytokines [12]. In these cases a cell-mediated response determines local changes with a release of specific cytokines and activation of Th2 lymphocytes (secreting IL-4, IL5, IL10 and IL13), that promote the production of IgE and amplify the inflammatory response (eosinophils, mast cells, neutrophils and natural killers chemotaxis), causing morphological and functional alteration of the mucosa [13,14].

Phenomena such as the increase of intestinal permeability and circulating immune complexes, can be associated with clinical symptoms in different organs and tissues, and this data are confirmed by the fact that increased intestinal permeability is reduced by effective elimination diet that excludes offending foods [15]. The higher incidence of food allergy in infants seems to be linked to the fact that infants are particularly prone to adverse reaction to cow’s milk proteins. In newborns the digestive enzymatic activity is not fully active and the secretory IgA system is not mature [16,17], The mucosa has an increased permeability shortly after birth [18]. For these reasons the passage of undigested proteins causing a primed immune response is facilitated. However, about 2% of the ingested food proteins are absorbed in an immunologically intact form also in adults [19].

### The immunopathogenesis of cow’s milk allergy

The immunological mechanism that lead to the development of cow’s milk allergy (or Cow’s Milk Protein Allergy- CMPA) is not still clarified. There are different mechanisms that contribute to the pathogenesis and the main two described mechanisms at the basis of this disease refer to IgE- and not-IgE- reactions [20]. Nevertheless, even if these two pathogenic pathways are the main described, there is a third mechanism causing CMPA, as a third group of symptoms attributed to cow’s milk allergy are unpredictably associated with IgE antibody (IgE-associated/cell-mediated disorders) [21].

IgE-mediated reactions are based on simply immunological mechanisms that are better identified than not-IgE-mediated ones. Since the onset of symptoms rapidly evolves (from several minutes to several hours after the contact with the allergen), this kind of mechanism is referred as “immediate hypersensitivity”. This mechanism developed in ancestors human beings to identify multicellular target parasites and to build up an immune response towards these organisms [22].

IgE-mediated CMAP is characterized by two stages: the first, of “sensibilization”, develops when the immune system is programmed in an aberrant way, so that IgE antibodies against cow milk proteins are secreted. These antibodies bind the surface of mast cells and basophiles, and the following exposure to milk proteins triggers the “activation” phase, when IgE associated to mast cells bind allergenic epitopes sited on milk proteins and unleash a rapid release of inflammatory mediators responsible for the allergic reaction. The allergens are ingested, processed and expressed by antigens presenting cells (APC) [23].

The interaction between APC and T lymphocytes promotes the modulation and the activation of B lymphocytes. These latter produce IgE antibodies that interact by their Fc portion with the allergen sited on mast-cells surface. The interaction among allergens on mast-cells/basophiles and IgE antibodies promotes an intracellular signalling process with a consequent cell degranulation and a release of histamine, PAF and other inflammatory mediators [24]. It is believed that both a deficiency in regulation and a polarization of milk-specific T cells toward type-2 T helper cells (TH2) lead to B-cell signaling to produce milk protein-specific IgE. [25,26]. Treg dysfunction plays a prominent role in lack of tolerance. It has also been shown that children outgrow their cow’s milk allergy in association with the development of Treg cells [27,28].

In some patients there is independent immunological tolerance to cow’s milk and exercise. However, anaphylaxis occurs when exercise followed intake of food (food dependent exercise-induced anaphylaxis) [29,30]. The exercise may induce serum hyperosmolality which increases histamine release [31] . Other possible explanations are that exercise may trigger the reaction since it decreases serum pH [32], or increases gastrointestinal permeability [33].

A high percentage of children and adults does not show circulating IgE specific for cow’s milk proteins and their skin prick test and serum specific IgE antibodies result negative. This occurs for the development of a not-IgE-mediated allergic disease. These reactions are characterized by a delayed set up, associated with the onset of symptoms after one hour or many days after the ingestion of cow’s milk proteins. For this reason, these reactions are classified as “delayed hypersensitivity”. However it is important to explain that the two reactions above described are not mutually exclusive and both can act in the same disease through different pathways [34].

The pathogenesis of non-IgE mediated reactions is supported by different theories: reactions mediated by Th1 cells, interactions between T lymphocytes, mast cells and neurons that alters the function of the smooth muscle and the intestinal motility [35].

It seems that there are discrepancies between the high number of natural resolution of manifestations linked to a not-IgE-mediated reaction during childhood and the predominance of these reactions in adulthood. This theory explains as these kind of reactions can appear later in life. On this regard, Zuberbier et al. observed patients of different age and they found a direct relationship between the incidence of not-IgE -mediated reactions and age. Nevertheless, other studies are needed to confirm this hypothesis [36].

### T lymphocytes and the induction of mucosal tolerance in CMPA

In food allergy the fine balance between mucosal tolerance and hypersensitivity is regulated by the immune system. This complex system includes molecules with regulatory properties, such as Transforming Growth Factor Beta 1 (TGF-beta1), IL-10 and Natural Killers. TGF-beta 1 and IL-10 are actually known as tolerogenic cytokines, produced by Treg, that are cells thought to regulate the immune system [37]. Also CD4 + CD25 + Foxp3+ T cells are described as important mediators for maintaining peripheral tolerance and suppressing the T lymphocytes proliferation [38,39].

A decreased immune response towards foreign antigens is defined as “mucosal or oral tolerance”, characterized by the deletion or suppression of T reactive specific-antigen cells and by production of T regulatory cells (Treg) that suppress the inflammatory response against benign antigens [39-44]. A dysfunction on Treg cellular activity seems to be a necessary background for the spread of both reactions (IgE- and not-IgE mediated CMPA), as the induction of mucosal tolerance in children is linked with an increase of Treg lymphocytes [45].

Actually, there are studies in progress with the aim to manipulate dendritic cells (specific cells presenting the antigen) as to improve the Treg function and/or to reestablish the Th1/Th2 balance and to promote the tolerance towards food antigens [46,47].

The Treg cells are about 5-10% of CD4+ T lymphocytes. They express the transcription factor “forkhead box” (FOXp3), that is a key gene for the generation and the maintenance of T lymphocytes [39]. To date, the involvement of Treg cells in the induction of oral tolerance is little known [48].

There are two distinct phases in the development of mucosal tolerance: the clonal deletion and the active suppression of the immune response, and it seems that Treg cells mediate both phases.

*In vivo* the depletion of anti-CD25 antibodies demonstrated that Treg play an important role in the oral sensitization to serum cow’s milk protein in mice (unpublished data by B. van Esch, B. Blokhuis, G. Hofman, L. Boon, J. Garssen, L. Knipples, L. Willemsen and F. Redegeld).

Further, Gri et al. demonstrated that Treg cells directly inhibit the mast cell degranulation through cell-cell contact and secretion of IL-10. *In vivo* depletion or inactivation of Treg cells causes enhancement of the anaphylactic response [40].

McNeil et al. investigated the role that Treg play in modulation of the human response. The authors showed that a previous treatment with the CD25 PC61 MoAb (monoclonal Antibodies) depleted a subpopulation of Treg in mice. In addition, PC61 seemed to alter the function of the remaining regulatory T cell population preventing their ability to modulate autoimmune diseases [44].

In conclusion, it seems that T lymphocytes subsets play a key role in allergic reactions to cow’s milk proteins, above all in those not-IgE mediated, and Treg cells are at the basis of oral tolerance to food allergy, so that an altered pattern of the immune system leads to all those atopic reactions, that are not explained by an IgE background [49].

### Probiotics and immune system: a new vision of the intestinal microbiota in CMPA

Toll like receptor (TLRs) recognize specific bacterial surface markers of microbiota, so called PAMP (Pathogen-associated molecular patterns) [50]. Some TLR agonists may activate Treg cells while others may trigger an allergic sensitization [51]. It could be possible that in industrialized societies, a decreased microbial exposure in early life leads to T-cell dysregulation which induce allergic disorders [52]. It is therefore, of great interest the characterization of probiotics bacterial strains that protect from sensitization to food allergens.

It is demonstrated that some chains of Lactobacillus and Bifidus Bacteria may influence the immune functions through different immunological mechanisms that act on enterocytes, antigen presenting cells, Treg and T and B effector lymphocytes [53-55].

In the gut, commensal bacteria allow a reduction of local inflammatory reactions. A single probiotic chain is necessary to maintain the integrity of the gut barrier reducing the effect of the antigen load. Some of these effects seem to be mediated by Treg, TLR9, TLR2 and TLR4. The intestinal microbiota also promotes the production of TNF-α and PGE2 that interfere with the development of tolerance mediated by dendritic cells [56]. As a matter of fact, an upregulation of TNF-α expression was found in gastrointestinal tract and in lesional skin of patients with acute urticaria [55]. In addition, most of the children with milk-induced cutaneous symptoms who showed an increased TNF-α secretion from milk-stimulated PBMCs were children with urticaria [57]. On this regard, other studies showed that probiotics directly potentiate the activity of human dendritic cells in promoting the polarization towards Th1. Evidences show that probiotics may promote the gut immune regulation and the allergenic tolerance [46].

In animal studies, probiotic supplementation seems to induce the development of Treg. *In vitro* human studies also suggest an increase of cytokine production (IL10) after ingestion of probiotics. This effect is limited only to certain species, such as L. Reuteri and L. Casei but doesn’t involve other species such as L. Plantarum. It is shown that L. Reiteri and L. Casei stimulate dendritic cells to increase the production of Treg cells. These latter produce high levels of IL10, inhibiting the activation of effector T lymphocytes [58,59].

These two lactobacilli are able to bind an intercellular lecitine-like adhesion molecule, specific for dendritic cells (know as non integrin grabbing molecule 3), blocking the interaction between the antibodies and these molecules [60,61]. Therefore probiotics show a positive effect in the induction of Treg cells.

The intestinal microbiota also influence the production of IgA in the distal intestinal tract. The gastrointestinal area plays a crucial role as integral part associated with the mucosal immune system. Currently it is widely described that B cells and effector T cells, sited in the gastrointestinal mucosa, develop also in the respiratory tract. This could explain why the intestinal microbiota can stimulate a systemic Th1 response.

It is unclear how probiotics can influence cell populations derived from bone marrow that do not pass through the intestine at any stage of maturation. In infant animals it is demonstrated that the presence of circulating monocytes mature in different ways depending on exposure to intestinal microflora. Many studies supporting the potential effects of the bone marrow showed that changes in gut microflora have a significant influence on the bone progenitor CD34cells release into circulation. Thus, a better understanding of the potential action of the intestinal microbiota is necessary for the assessment of possible causal effects and the role of probiotics as agents for prevention and therapy of atopic diseases, such as CMPA.

## Competing interests

All authors declare not to have any competing interest.

## Authors’ contribution

All authors declare to have equally contributed to the writing of the paper, to the collection of literature references and to the building up of the paper, as far as the revision of the paper. All authors read and approved the final manuscript.
