# Correction: The immunopathogenesis of cow’s milk protein allergy (CMPA)

**DOI:** 10.1186/1824-7288-39-82

**Published:** 2014-01-28

**Authors:** Giovanna Vitaliti, Carla Cimino, Alfina Coco, Andrea Domenico Praticò, Elena Lionetti

**Affiliations:** 1 Bronchopneumoallergology and Cystic Fibrosis O.U., Departement of Pediatrics, University of Catania, AOU Policlinico-OVE, Via Santa Sofia 78, 95123, Catania, Italy

## Correction

After publication of the original article [1] it came to the publishers attention that the authors’ first and last names have been inadvertently transposed. The correct authors’ list should read as indicated above. We apologize for any inconvenience this has caused.
